# A rare case of plasmacytoma of the ovary: a case report and literature review

**DOI:** 10.3332/ecancer.2013.288

**Published:** 2013-01-15

**Authors:** PN Shakuntala, SR Praveen, B Shankaranand, K Rajshekar, K Umadevi, UD Bafna

**Affiliations:** 1 Department of Gynaecologic Oncology, Kidwai Memorial Institute of Oncology, Dr. M.H. Mari Gowda Road, Bengaluru 560029, Karnataka, India; 2 Department of Pathology, Kidwai Memorial Institute of Oncology, Dr. M.H. Mari Gowda Road, Bengaluru 560029, Karnataka, India

**Keywords:** ovarian plasmacytoma, CA-125, LDH, optimal cytoreductive surgery, carboplatin

## Abstract

**Objective:**

Extramedullary plasmacytomas are rare. Ovarian plasmacytomas, for which treatment options vary, are more unique and even more rare. We will consider the option of optimal cytoreductive surgery followed by adjuvant chemotherapy consisting of carboplatin (AUC-2) to prevent massive ascites and pleural effusion.

**Case report:**

We report a case of ovarian plasmacytoma in a 35-year-old woman presenting with abdominal pain due to the mass. She underwent optimal cytoreductive surgery. A post-operative histopathologic diagnosis of ovarian plasmacytoma was confirmed. She was assigned stage IIC disease. She received three cycles of single agent carboplatin for rapidly refilling ascites and pleural effusion. Her response was dramatic. There is no evidence of recurrence clinically for more than 14 months. The patient is receiving follow-up care.

**Conclusion:**

Multimodality treatment comprising of optimal cytoreductive surgery followed by carboplatin-based chemotherapy is a novel observation and may be an option for the treatment of these rare tumours. This options needs to be further researched.

## Introduction

Plasmacytoma results from clonal proliferation of plasma cells that are identical to plasma cells of myeloma on both the cyotologic and immunophenotypic levels. Plasmacytoma can be subclassified as an osseous disease or an extraosseous tumour [1].

Plasmactyoma exists in three clinical forms: multiple myeloma (MM), medullary plasmacytoma (MP), and extramedullary plasmacytoma (EMP) [2]. The EMP is a localized collection of tumour cells in the soft tissue with plasma cell differentiation, but without evidence of MM, MP or lymphoma [2]. The adjective “plasmablastic” is used when there are more than 30% of plasmablasts in routine sections [3]. We have reviewed seven cases in the literature [4–10], and due to a paucity of information there is no evidence based consensus on adjuvant therapy in these women. 

## Case report

**Figure 1:**
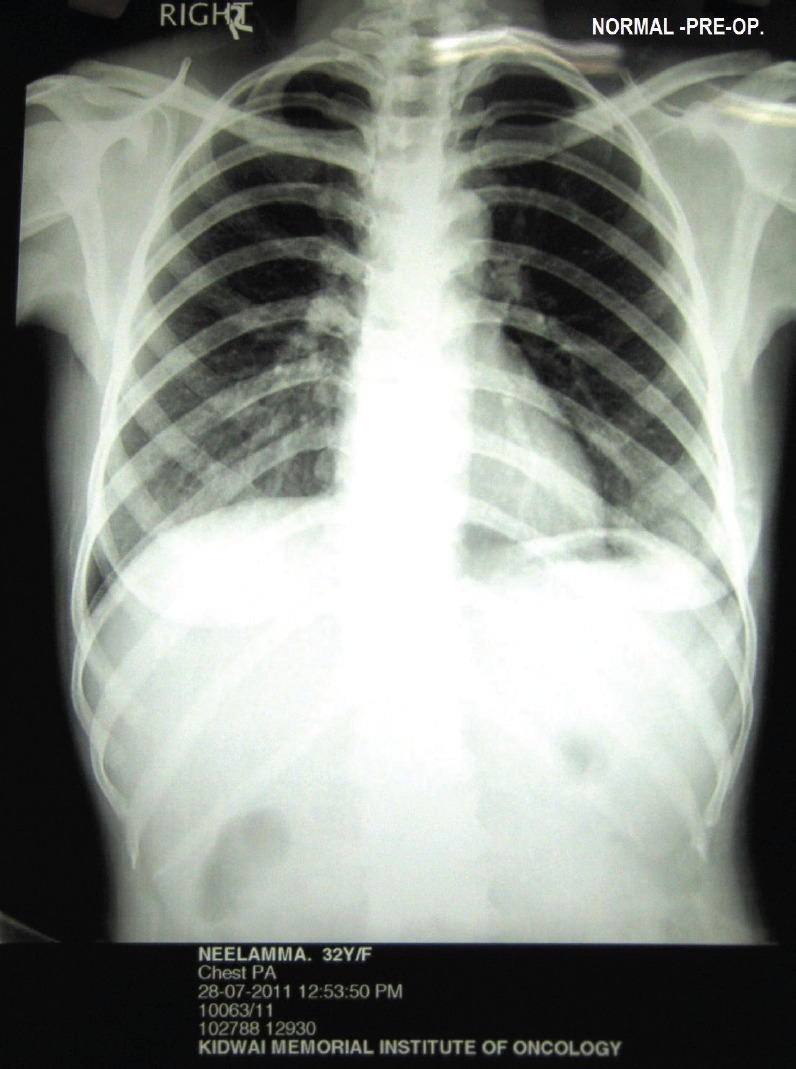
Normal pre-operative chest X-ray

**Figure 2:**
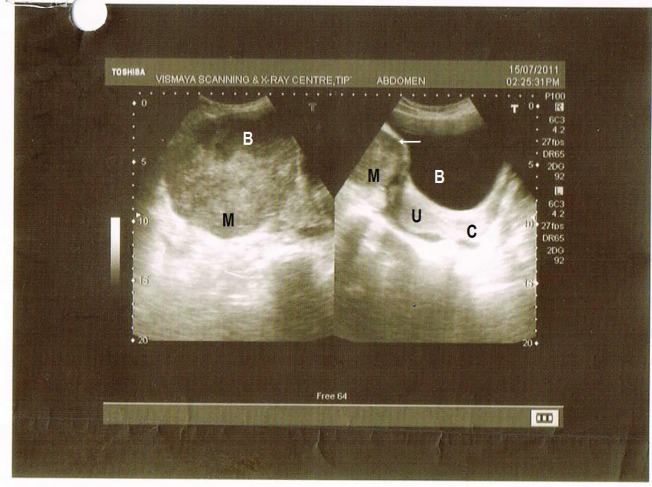
Right side of picture shows normal bladder (B), white arrow denotes the mass (M) indenting the bladder, U-normal uterus, and C-normal cervix

A 35-year-old multiparous woman presented with a history of abdominal mass associated with acute episodes of pain for 15 days. She had severe pallor. On vaginal and rectal examination, the uterus and cervix felt normal. A mobile abdomino-pelvic mass measuring 15 × 15 × 15 cm arising from the right adnexa, occupying the anterior fornix and extending to the left iliac fossa and lumbar region was identified. There was tenderness during examination. Bilateral parametria were supple and rectal mucosa was free. Except for a haemoglobin level of 8 gm%, the remainder of the haemogram, serum biochemistry, chest X-ray (Figure 1), serum anti-HIV, and anti-HbSAg levels were normal. Serum cancer antigen (CA-125) 178 U/ml and lactic dehydrogenase (LDH) 899 U/l were high. Beta human chorionic gonadotrophin (**β**-hcg), carcinoembryonic antigen (CEA), and alpha fetoprotein levels (AFP) were normal. An ultrasound revealed the presence of a solid heterogeneous mass measuring 10.9 × 10.8 cm, with increased vascularity on colour Doppler (Figure 2).

**Figure 3:**
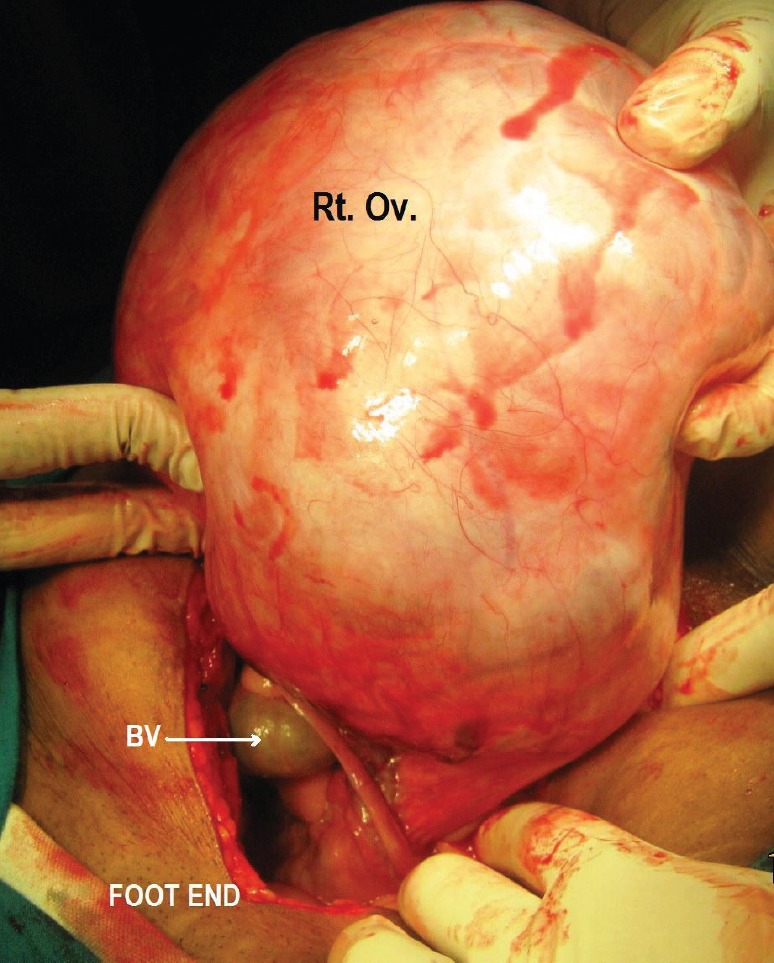
Showing intraoperative, (Rt. Ov.) is the right ovarian tumour appearing solid in consistency and (BV) are the tortuous blood vessels supplying the tumour

**Figure 4:**
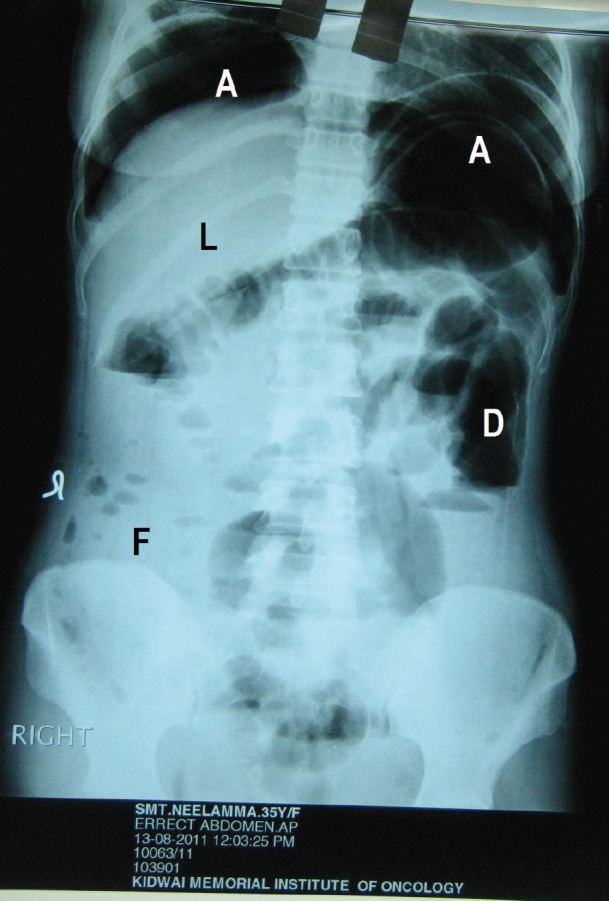
Post-operative erect abdominal X-ray showing air fluid levels under diaphragm (A), (L) liver, (D) dilated large bowel without fluid level, (F) ascitic fluid in the peritoneal cavity

**Figure 5:**
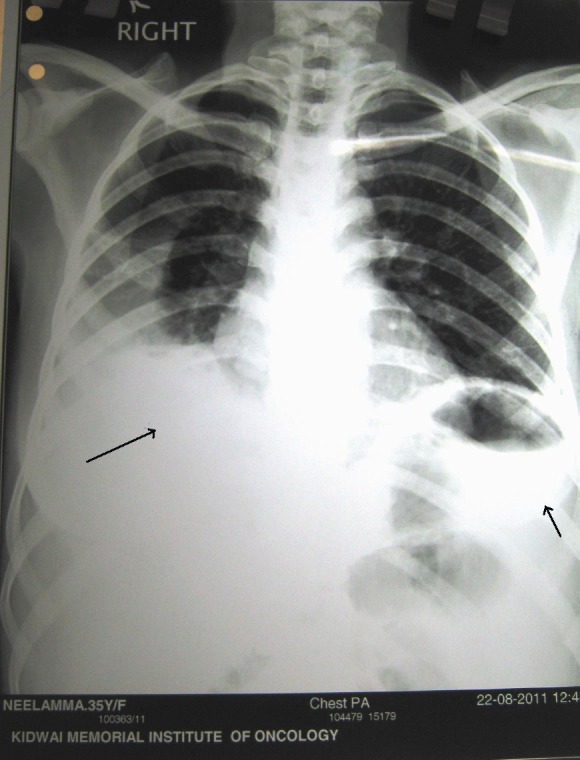
Post-operative chest X-ray showing bilateral pleural effusion right more than left (following pleural tapping)

**Figure 6:**
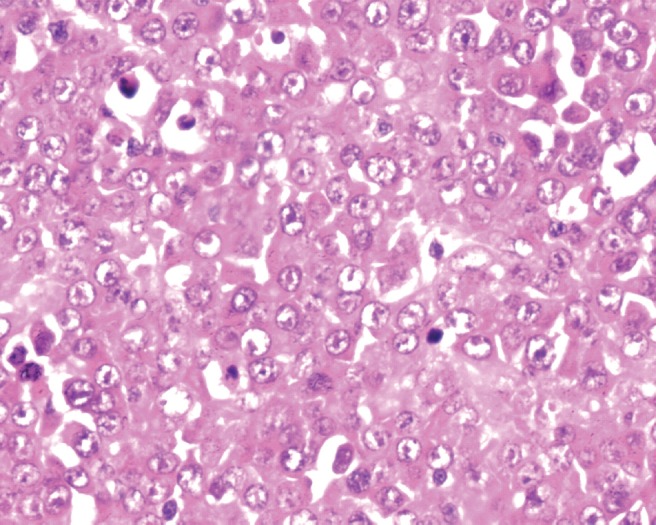
Sheets of plasmablasts with eccentric nuclei, prominent nucleoli, and abundant eosinophilic cytoplasm (H&E, 40×)

**Figure 7:**
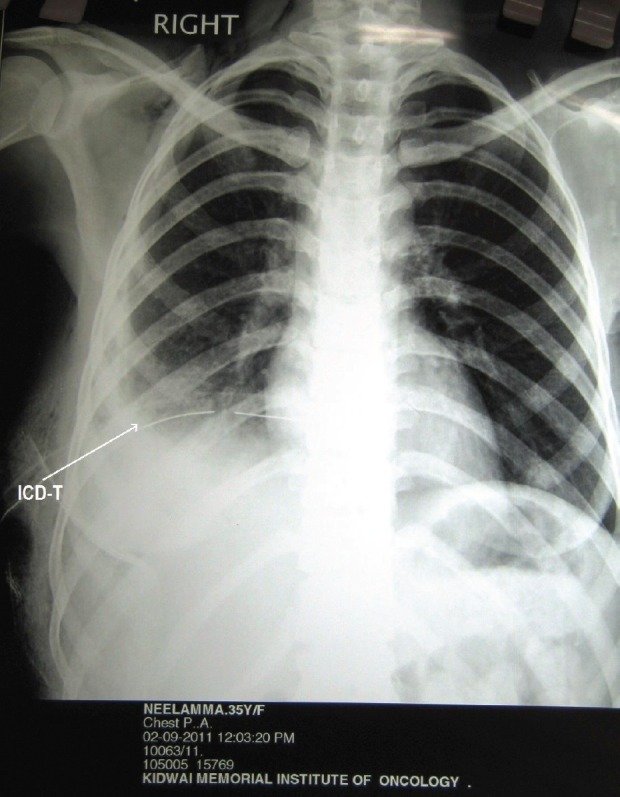
Following carboplatin, resolving pleural effusion with intercostal drainage tube (ICD-T) *in situ* and resolving ascites

During surgery, minimal haemorrhagic ascites was noted. A very vascular right ovarian mass measuring 14 × 15 × 6 cm, with solid cystic areas adherent to the bladder peritoneum along with deposits on it, was seen. The uterus, left tube and ovary, appendix, omentum (nodular), and upper abdominal viscerae were normal. Bulky nodes were palpable along the paraaortic and iliac regions (Figure 3). An intraoperative frozen section revealed the presence of poorly differentiated neoplasm carcinoma. An extrafascial hysterectomy with bilateral salpingo-oophorectomy followed by bilateral pelvic lymphadenectomy, paraaortic lymph node dissection, excision of deposits on the bladder peritoneum, and total omentectomy was performed. The patient went through a very stormy post-operative period with repeated and rapidly refilling ascites, as well as right-sided pleural effusion. She also had paralytic ileus. She was treated symptomatically with blood and blood products, timely paracentesis, and pleurocentesis (Figures 4 and 5). Her general condition deteriorated due to repeated pleural effusion, and hence an intercostal drainage tube was inserted to drain the fluid. She continued to produce pleural effusion whose cytology was reactive mesothelial cells, with no evidence of malignancy. The histopathology was inconclusive depicting the presence of poorly differentiated neoplasm of the right ovary and the bladder deposits. The omentum, all the pelvic (12 nodes) and paraaortic [7] lymphnodes were reactive. She was allotted FIGO Stage IIC. Immunohistochemistry (IHC) was requested for appropriate categorization (Figure 6). Since her clinical condition was not improving, single agent weekly carboplatin, dose of area under curve-2 was administered hoping to reduce the pleural and ascetic fluid collection. She responded dramatically. By the seventh post-chemotherapy day, the pleural fluid collection was almost nil (Figure 7). She received three more cycles of weekly carboplatin. Ca-125 and LDH had normalised. 

**Figure 8:**
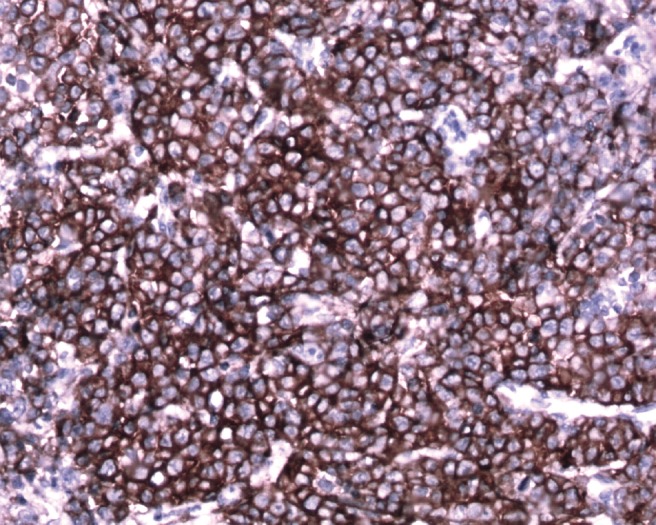
Immunohistochemical staining with CD138 shows strong membrane positive plasmablasts (brown pigment) (20×)

Immunohistochemistry depicted a plasmatoid neoplasm positive for CD-138, lambda, CK, and focal positivity for EMA and negative for CK7, CD99, Inhibin, CD117, Synaptophysin, Chromogranin, Mic2, LCA, HMB45 CK20, SMA, Desmin, CD34, S100, CD79a, and kappa. Possibilities of plasmablastic lymphoma and plasmacytoma were considered (Figure 8). She declined further myeloma work, and hence the patient has not received bone marrow or M protein detect, and was not willing to receive any further adjuvant treatment in the form of radiotherapy due to financial constraints. She is on follow-up care for 14 months and there is no clinical evidence of disease. 

## Discussion

Extramedullary plasmacytoma (EMP) is a very rare primary soft tissue plasma cell tumour, most commonly (90%) occurring in the upper aero digestive tract. They constitute fewer than 5% of all plasma cell tumours, generally remain localized, and are more responsive to therapy [1, 2]. We report this case for its rarity and several clinical, diagnostic dilemmas, and treatment related challenges. 

A pre-operative diagnosis of stromal tumour of the ovary was considered as the patient was aged 35 years presenting with an acute onset of lower pain abdomen, a solid pelvic mass without ascites and elevated CA-125 levels 170 U/ml and LDH 899 U/l. Beta human chorionic gonadotrophin (β-hcg), carcinoembryonic antigen (CEA), and alpha fetoprotein levels (AFP) were normal. She was not immunocompromised, her HIV and HbSAg status were negative. On reviewing the literature (Table 1), most of the women presented with a mass and pain in their abdomen. Zhong *et al *[11] reported a lady with intraperitoneal haemorrhage (ovarian rupture) similar to the present case who also had a mass with intermittent pain in her abdomen and haemorrhagic ascites. There is no mention of the associated elevated CA-125 levels or the LDH levels (Table 1). Post-operatively both the values had normalised. Since the patient declined further investigations and treatment due to financial constraints, we are unable to comment on the role of these markers in disease monitoring.

Due to heterogeneity in the reports, a certain inconsistency in evaluations does occur but it is to be noted that the left ovary was more common, involved in four cases, and the right side was involved in two cases, including the present case. All women presented with a mass in their abdomen without ascites, and the size of the masses was greater than 12 cm. Serum immunoelectrophoresis was performed in four of the seven patients, with IgG paraprotein positive in three cases, IgA positive in one, and one without a monoclonal protein detected (Table 1). Various standards have been used by different authors regarding immunohistochemical staining. In the present case, there was plasmacytoid neoplasm positive for CD138, lambda, CK, and focal positivity for EMA. All the seven cases were early stage disease as reported by Emery *et al *[10]. The present case was a stage IIC disease. Atypicality lies in recurrent and rapid fluid accumulation in the abdomen and pleural spaces. Repeated paracentesis and abdominocentesis were performed to relieve her symptoms of dyspnoea and pain in her abdomen. Finally, she was relieved of her symptoms with the placement of an intercostal drainage tube (ICD-tube) and on starting weekly single agent carboplatin with area under curve (AUC) of 2. Due to the above dilemmas regarding the deteriorating general condition, rapidly refilling pleural effusion, ascites, and histopathology being inconclusive other than a poorly differentiated neoplasm (stage IIC), a final decision to start on weekly single agent carboplatin was made. There was a dramatic response. Her ICD-collection reduced from 1600 ml to almost nil by the seventh post-chemotherapy day. She received three more cycles of weekly carboplatin which completely resolved pleural effusion and ascites, her general condition improved. Ca-125 was 17.7 U/ml and LDH-322 U/l at the end of chemotherapy. Due to financial constraints, the woman declined further myeloma work and only came for follow-up care. She has no evidence of disease clinically for the last 12 months. These unusual complications in plasmacytoma of the ovary have not been reported so far. The dramatic response to carboplatin is a novel observation. To date there is no consensus regarding post-operative adjuvant therapy in extramedullary plasmacytoma of the ovary. The rate of progression and survival data are very sparse and are collected from case reports (Table 1). Since most of them were early stage disease, the survival periods have ranged from 3 to 24 months and beyond. The patient who died at 3 months is exceptional; she had multi-organ failure and died [5], (Table 1). 

## Conclusion

Due to a paucity of information with these exceedingly rare tumours, it is essential to consider them in the differential diagnosis of women in the reproductive age group, who present with abdominal masses without ascites. Post-operative ascites and pleural effusion is an area of concern which raises the issue of adjuvant treatment. The role of single agent carboplatin in extramedullary plasmacytoma of ovary as an adjuvant treatment needs to be explored. 

**Authors’ contributions: **Dr. PN Shakuntala was involved in manuscript preparation of the observations, patient care, reference hunting, and final editing. Dr. K Rajshekar was involved with patient care, editing the manuscript. Dr. B Shankaranand was concerned with the histopathologic and immunohistochemical diagnosis. Dr. UD Bafna played a key role in contemplating the novel therapy of using carboplatin. Dr. SR Praveen was involved with final editing and reference hunting, and Dr. K Umadevi was involved with patient care and manuscript editing.

## Figures and Tables

**Table 1.** 

Author	Age/ symptoms	Ovarian size/ side	Ig	CA-125 U/ml and LDH U/L	Post op. CT	F/U	Follow up status
Voegt [4] 1938	30 — —	“fist”	NA	NA	NA	NA	NA
Bambirra [5] 1984	44, abd**. pain bilateral	R 14.3 × 5.3 × 4 cm L 12.3 × 9 × 36 cm	IgG	NA	NA	3 mon.	DOD
Hautzer [6] 1984	56, abd. mass	left 24 × 31 × 14.3 cm	IgG	NA	NA	NA	NA
Talerman [7] 1987	35 .abd. mass	unilateral 15.3 × 12.3 × 9 cm	NA	NA	NA	9 mon.	NA
Cook [8] 1988	63 ,abd. pain	left 12.3 × 10 × 3.7 cm	IgA	NA	NA	24 mon.	NA
Andze [9] 1993	12 ,pelv. mass	left 12.3 × 8 × 3.8 cm	Negative	NA	NA	11 mon.	NA
Emery JD [10] 1999	54 ,abd. swell	left 15.3 × 13 × 3.8 cm	IgG	NA	NA	24+ mon.	NED
Zhong YP et al 2012 [11]	54, abd pain	right cystic ovary (12 cm × 12 cm × 10 cm).	MM IgA-. type III stage A	NA	Proposed- bortezomib and dexamethasone, etoposide, cyclophosphamide, and cisplatin (DECP)	NA	NA
Present case 2012	35, abdominal mass, intermittentpain	right 14 × 13.5 × 6 cms	Not done	178 and 899.	Weekly carboplatin AUC-2	Post op. Ca-125 (17.7U/ml) 14 months	NED

** * abd* abdominal, *pelv* pelvic, *Ig* immunoelectrophoresis immunoglobulin, *B–J* Bence Jones protein, *L* left, *R* right, *F/U* follow-up, *AWD* alive with disease, *NED* alive with no evidence of disease, *DOD* dead of disease, *NA* not available, *neg* negative, *Post OP* post-operative, *CT* chemotherapy
